# Editorial: *Nucleic Acids Research* annual Web Server Issue in 2014

**DOI:** 10.1093/nar/gku629

**Published:** 2014-07-01

**Authors:** Gary Benson

**Affiliations:** Executive Editor, Web Server Issue, Nucleic Acids Research

The 2014 Web Server Issue of Nucleic Acids Research is the 12th in a series of annual special issues dedicated to web-based software resources for analysis and visualization of molecular biology data. It is freely available online under NAR's open access policy. The present issue reports on 80 web servers.

**Topics.** This year's special emphasis is on tools for synthetic biology design, analysis of high throughput sequencing data, network and pathway analysis, and biological text mining. A total of 19 papers deal with these topics. The other major topic categories include analysis involving DNA and RNA (12 papers); gene prioritization (5 papers); drug-target interactions (5 papers); and proteins, including structure, ligand binding, and functional site prediction (24 papers).

The 2014 Web Server issue continues the presentation of two special categories, one for stand-alone programs that analyze high-throughput data, such as high throughput sequencing data, and one for large collections of web services for automated analyses that can be utilized programmatically rather than through manual interaction with a web browser. Six papers fall in these categories.

**Acknowledgements.** The Web Server issue would not be possible without the work of the many scientists and programmers who have provided us with outstanding, freely available web resources, and the conscientious efforts of literally hundreds of reviewers.

**Figure 1.**
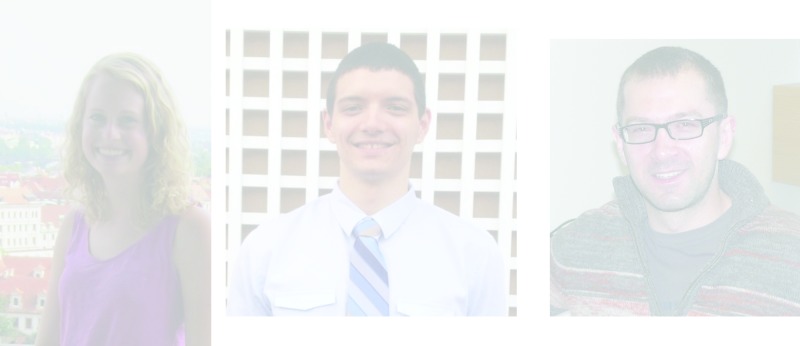
Allyson Byrd and Joe Perez-Rogers, PhD students in the Boston University Bioinformatics Graduate Program, and Artem Mamonov, Research Associate in the BU Department of Biomedical Engineering, helped with testing the Web Server proposal websites.

My work was made possible, first, by the editorial assistance of Fay Oppenheim. Thank you. Thanks also to Allyson Byrd and Joe Perez-Rogers, PhD students in the Boston University Bioinformatics Program, and Artem Mamonov, Research Associate in the BU department of Biomedical Engineering, for their outstanding assistance in evaluating the proposal websites (Figure 1). Additional thanks to Martine Bernardes-Silva, Editorial Manager, NAR, and Jennifer Boyd and the staff at Oxford University Press.

**Instructions for Submissions.** To streamline the review process, authors are required to send a one-page summary of their web server to the editor, Dr. Gary Benson (narwbsrv@bu.edu), for pre-approval prior to manuscript submission. For the 2014 issue, 269 summaries were submitted and 106, or 39%, were approved for manuscript submission. Of those approved, 80, or 75%, were ultimately accepted for publication.

Review of a summary includes evaluation of the proposal and extensive testing of web server functionality. The key criteria for pre-approval are high scientific quality, wide interest, the ability to do computations on user-submitted data, and a well designed, well implemented, and fully functional website. Note that there is a minimum two-year interval before publication in the Web Server issue for web servers, or essentially similar web servers, that have been the subject of a previous publication, including publication in journals other than NAR.

With respect to the website, the following are guidelines for approval.

1. It should have an easy-to-find submission page with a simple mechanism for loading test data and setting test parameters. The preferred method is one-click loading. Additional mechanisms that assist the user in submitting data should be implemented where appropriate, for example, automatic download of a pdb structure file once the user has entered the appropriate identifier.
2. Output should be dynamic and rich in detail. Wherever possible, supporting evidence used in calculations and/or links to external databases containing additional information should be provided. Numerical, textual, and visual output should be mixed and any visualization tools that add information or increase the user's understanding should be utilized. Note that output consisting merely of a few numerical values, a static spreadsheet, or a series of files to be opened in other programs will not be approved.
3. Web servers that do not finish their calculations immediately must implement a mechanism for returning results to the user. Notification by email may be provided as an option, but an alternative that returns a web link at the time of data submission, which the user can then bookmark and access at a later time, is required. This link should ideally report the status of the job (queued, running, finished). Websites that require a guest login will not be approved. Note that uploaded data and the results of analysis for each user must be private and not viewable by other users.
4. The website should be supported by an extensive help section or tutorial that guides the user through the submission process, contains details about input file formats and parameters, and importantly, explains the meaning of the output. Whenever possible, the help pages should link to dynamic output examples similar to those provided by the website. Text and figure help pages, rather than video tutorials, are preferred because they simplify quick look-up.
5. Any proposal for a web server that is *predictive* must include details on validation of predictions from new data not used in training. N-fold cross validation methods will not be considered sufficient. Details should include size and composition of the validation data set (number of positive and negative cases), and several measures of predictive performance, including sensitivity, specificity, and precision. Proposals are frequently rejected for lack of adequate prediction validation information.
6. Websites not clearly designed to accept and analyze user-submitted data will be rejected. This applies to those established primarily for lookup or exploration in a data set, or serve the function of “data aggregators.” Authors of websites that provide novel data should consider the NAR Database Issue as a possible venue (see the instructions at http://www.oxfordjournals.org/our_journals/nar/for_authors/msprep_database.html).
7. Proposals that describe a new analysis method are generally not appropriate for the Web Server issue because limited space makes adequate method description and validation problematic. Authors of such methods might instead consider sending their manuscript to NAR as a regular computational biology paper (see the instructions for authors at http://www.oxfordjournals.org/our_journals/nar/for_authors/criteria_scope.html#Computational%20Biology).

**Special Emphasis for 2015.** For the 2015 issue, the topics of special emphasis will be tools for synthetic biology design, analysis of high throughput sequencing data, and network and pathway analysis.

**Deadlines for 2015.** Authors wishing to submit manuscripts for the 2015 Web Server issue must submit their one page proposal along with the URL address of the fully functional website to narwbsrv@bu.edu by 31 December 2014. Detailed instructions and requirements are presented at http://www.oxfordjournals.org/nar/for_authors/submission_webserver.html. This information should be consulted before sending in the summary. The deadline for submission of articles is 31 January 2015.

**Requirement for References Links.** Manuscripts submitted for the 2015 issue must format their References section to include active links to electronic versions of the cited papers, including links to PubMed, PubMed Central, and a DOI link. Instructions for incorporating these links into the manuscript are presented at http://www.oxfordjournals.org/nar/for_authors/submission_webserver.html.

